# Supplementary material for: Pleural Mesothelioma Diagnosis for the Pulmonologist: Steps Along the Way
Source: Cancers (Basel). 2025 Dec 1;17(23):3866. doi: 10.3390/cancers17233866 (PMC12691341; doi:10.3390/cancers17233866)
Supplement: Supplementary file 1 [file cancers-17-03866-s001.zip › cancers-3998018-supplementary.pdf]

Table S1. *Search strategy used for the databases.*

(Accessed by using University of Udine - VPN). Search performed on: 1 June 2025

Databases	Group	Search Syntax	Records
**PubMed**	1	("Mesothelioma"[Mesh] OR "pleural mesothelioma"[tiab]) AND ("Diagnosis"[Mesh] OR diagnosis[tiab] OR "diagnostic pathway"[tiab] OR "diagnostic steps"[tiab] OR "diagnostic approach"[tiab])	**1575**
**Scopus**	1	(TITLE-ABS-KEY ("mesothelioma" OR "pleural mesothelioma")) AND (TITLE-ABS-KEY ("diagnosis" OR "diagnostic pathway" OR "diagnostic steps" OR "diagnostic approach")) AND PUBYEAR > 2004 AND PUBYEAR < 2026 AND (LIMIT-TO (LANGUAGE , "English")) AND (LIMIT-TO (EXACTKEYWORD , "Human")) AND (LIMIT-TO (DOCTYPE , "ar")) AND (LIMIT-TO (SUBJAREA , "MEDI") OR LIMIT-TO (SUBJAREA , "PHAR") OR LIMIT-TO (SUBJAREA , "HEAL") OR LIMIT-TO (SUBJAREA , "MULT") OR LIMIT-TO (SUBJAREA , "ENVI"))	**3414**
		**Total evaluated**	**4989**

Selection criteria:

- Time range: 01/01/2005 - 01/06/2025;
- Language: English;
- Species: Humans;
- Subject area: Medicine, Healtcare, Health Professionals, Pharmacology, Toxicology, Pharmaceuticals, Multidisciplinary, Environmental Science
- Source type: journals;
- Type of study: systematic reviews, meta-analyses, randomized control trials, original research papers and case reports.

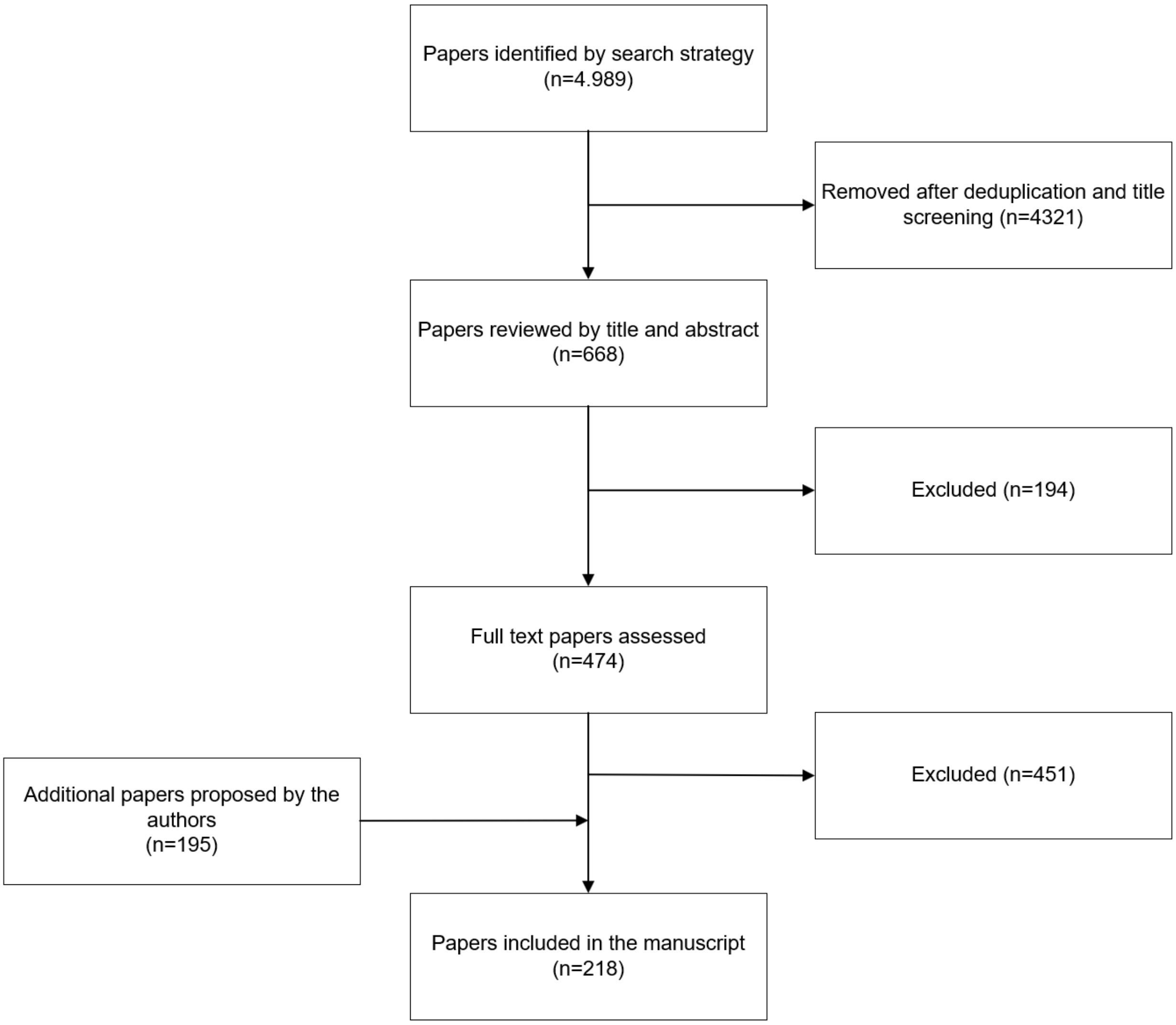

Figure S1. *Consort flow diagram of the review process.*
